# New Strategies in the Treatment of Ta-T1 Transitional Cell Carcinoma (TCC) of the Bladder

**DOI:** 10.1100/tsw.2006.406

**Published:** 2006-10-02

**Authors:** Maurizio A. Brausi

**Affiliations:** AUSL Modena, Italy

**Keywords:** superficial bladder cancer

## Abstract

In recent years, there have been many advances in the treatment of superficial bladder cancer. Standard intravesical chemotherapeutic agents can now be delivered more effectively thanks to new technological advances in drug delivery. Local microwave hyperthermia and electromotive drug administration are of particular interest. Research has also shown that different combinations of drugs and sequential drug delivery of two or more different drugs for differing periods of time also increase the effectiveness of possible treatments of superficial bladder cancer. Furthermore, new chemotherapeutic drugs for intravesical use are being investigated in various clinical trials, with gemcitabine showing particularly promising results. Also in the pipeline are new approaches to treatment such as gene therapy, but these will need to be developed much more before they become part of routine practice.

## TECHNOLOGICAL ADVANCES IN INTRAVESICAL CHEMOTHERAPEUTIC DRUG DELIVERY

Better delivery of standard intravesical chemotherapeutic agents has the potential to improve outcome in high-risk patients. Currently there are two competing technologies that are being investigated in clinical trials that may result in a resurgence in the use of intravesical chemotherapy.

Local microwave hyperthermia in conjunction with mitomycin C (20 mg/50 cc) was compared in a multicenter, randomized trial to intravesical mitomycin C alone in 83 patients[1]. Hyperthermia was delivered at a temperature of 42°C for at least 40 min.

At a minimum follow-up of 24 months, there was a statistically significant reduction in recurrences between the two groups (17.1% for the chemothermotherapy vs. 57.5% for chemotherapy alone). This modality has also been used in treating patients with high-grade superficial bladder cancer (Ta T1 G3) as a prophylactic (40 mg MMC) or ablative (80 mg MMC) protocol by Gofrit et al.[2]. In 24 patients administered the prophylactic protocol, 62.5% were recurrence-free after a mean follow-up of 35.3 months. The ablative protocol was administered to 28 patients with complete ablation of the tumor in 75% of cases and a recurrence-free rate of 80.9% at a mean follow-up of 20 months.

Electromotive drug administration (EMDA) has beeen proposed to improve drug delivery across biological membranes with increased accumulation in bladder tissue. In a phase II trial with a marker lesion, Brausi et al. showed that EMDA of MMC was effective in Ta T1 TCC of the bladder[3]. After this, Di Stasi et al. randomized three groups of patients with CIS (carcinoma *in situ*) to 40 mg eMMC (electromotive MMC) instillation with 20 mA electric current for 30 min, 40 mg passive MMC with a dwell time of 60 min, or 81 mg BCG (bacillus Calmette-Guerin) with a dwell time of 120 min[4]. Patients were scheduled for an initial six weekly treatments, a further six weekly treatments for nonresponders, and a follow-up ten monthly treatments for responders. There was a statistically significant superior complete response rate at 6 months for eMMC (58%) compared to passive MMC (31%). The response rate of the eMMC approached that of BCG (64%). Peak plasma MMC was significantly higher following e MMC than after passive MMC (43 vs. 8 ng/ml), supporting the hypothesis that electromotive MMC increases tissue levels.

## SEQUENTIAL DRUG THERAPY

Alternative approaches have been investigated to improve the efficacy of intravesical therapy. Several investigators have explored the use of BCG combined with chemotherapy. The rationale for giving chemotherapy prior to BCG is to induce sloughing of the urothelium, allowing BCG to better interact with fibronecting and initiating an immune response. However, BCG *immediately* after epirubicin is not well tolerated. Erol et al. reported that over a third of patients discontinued treatment due to severe cystitis[5].

Rintala et al. reported a novel approach of alternating mitomycin C and BCG for prophylaxis of superficial papillary bladder cancer[6]. After an induction course of mitomycin C, 188 patients with recurring Ta and T1 tumors were randomly assigned to maintenance mitomycin C vs. alternating mitomycin C and BCG instillations (Pasteur strain). The patients were treated for 2 years and the mean follow-up was 34 months.

Alternating mitomycin C and BCG was equal in efficacy to mitomycin C and was clearly superior to TUR (transurethral resection) alone. For CIS patients, Rintala et al. reported that alternating BCG with mitomycin C was superior to mitomycin C[7]. The superiority of BCG and mitomycin C could, however, be attributed to the presence of BCG in one arm and not the other rather than the alternating mode of delivery. More importantly, no significant side effects developed in the alternating group. A different randomized prospective trial of sequential BCG and eMMC was reported by Di Stasi et al.[8]. Compared to 6-weekly BCG followed by 1 year of monthly maintenance, a program of (BCG, BCG, eMMC) × 3 for weekly induction and (eMMC, eMMC, BCG) × 3 for monthly maintenance resulted in a statistically significant decrease in recurrence at 5–6 years from 47 to 28%. In contrast, Kaasinen et al. report that alternating BCG with interferon-alpha after an induction course of mitomycin C was inferior to BCG after mitomycin C[9].

A randomized phase III study of intermediate and high-risk superficial bladder cancer comparing sequential mitomycin C for 4 weeks followed by weekly BCG vs. weekly mitomycin C showed no difference in recurrence or progression[10]. BCG alternating with epirubicin was not superior to BCG alone, but had fewer side effects[11].

BCG monotherapy was superior to an induction course of mitomycin C followed by alternating mitomycin C and BCG instillations in patients with CIS disease[12]. The recurrence-free survival and progression-free survival were also superior in the patients treated with BCG only.

Serretta et al. have reported using adjuvant sequential mitomycin C and epirubicin in 91 out of 137 patients with T1G3 bladder cancer after initial TUR[13]. With close to 20 years of follow-up, the overall recurrence rate was 51%, but significantly less in the sequential chemotherapy protocol. The overall progression rate of 9.5% was no different. Only 7% of patients died of bladder cancer and the cystectomy rate was also 7%.

As yet, it is not possible to determine whether mixed chemotherapy or chemoimmunotherapy programs will dependably result in improved outcomes.

## NEW CHEMOTHERAPEUTIC DRUGS FOR INTRAVESICAL USE

Gemcitabine (2’,2’-difluoro-2’ deoxycytidine) is a novel deoxycytidine analog with a broad spectrum of antitumor activity. Gemcitabine has a molecular weight of 299.66 and, after intracellular activation, the active metabolite is incorporated into DNA, resulting in inhibition of further DNA synthesis. Gemcitabine may also inhibit ribonucleotide reductase and cytidine deaminase as part of its cytotoxic activity[14]. Unlike most other chemotherapeutic agents, gemcitabine has no vesicant (tissue irritating) properties, suggesting it may be better tolerated in the bladder.

Gemcitabine is highly effective (overall response rates ranging from 22.5 to 28%) and well tolerated as both first- and second-line, single-agent therapy for the treatment of metastatic TCC[15,16]. Studies have reported a low incidence of systemic side effects. A randomized, multicenter, phase III study demonstrated that patients with unresectable or metastatic disease treated with gemcitabine plus cisplatin (GC) had a similar rate of survival to patients treated with MVAC (methotrexate, vinblastine, doxorubicin[Adriamycin], and cisplatin), and GC had a better safety profile and tolerability[17]. Based on its excellent clinical activity, patient tolerability, and chemical characteristics, gemcitabine represents a logical candidate for intravesical therapy.

Dalbagni et al.[18] reported a phase I study of intravesical gemcitabine twice a week for 3 weeks, followed by a second cycle after a week of rest, in a heavily pretreated population with BCG – refractory TCC. This study demonstrated that intravesical gemcitabine was well tolerated with minimal bladder and acceptable myelosuppression. Serum levels of gemcitabine were undetectable at concentrations of 5, 10, and 15 mg/ml. However, serum gemcitabine was detected at a concentration of 20 mg/ml. Complete response (CR), as defined by a negative post-treatment cystoscopy including a biopsy of the urothelium and a negative cytology, was achieved in 7 out of 18 patients (39%)[18]. This was followed by a phase II study of patients with BCG refractory TCC to determine the efficacy of gemcitabine as a intravesical agent. Twenty-eight patients completed therapy and 16 achieved a CR[19].

Laufer et al.[20] reported a phase I study of weekly intravesical gemcitabine in 15 patients who received prior intravesical therapy. Serum gemcitabine levels were undetected at concentrations of 5, 10, 15, and 20 mg/ml, while low concentrations were present in all patients receiving 40 mg/ml. However the metabolite dFdU (2’2’-difluorodeoxyuridine) was detectable in plasma of patients receiving gemcitabine at concentrations of 15 mg/ml or higher, implying minimal absorption of gemcitabine at lower doses. The authors concluded that intravesical gemcitabine is well tolerated, with minimal toxicity. Furthermore, no evidence of recurrence at 12 weeks was noted in 9 out of 13 evaluable patients[20].

In a recent phase I study, De Berardinis et al.[21] reported nonsystemic detection of gemcitabine at a concentration of 40 mg/ml. However, the inactive metabolite was detected in plasma. They were able to demonstrate the activity of deoxycytidine kinase in tissue samples, an enzyme that produced 2’,2’-difluoro-deoxycytidine triphosplosphate, the active metabolite of gemcitabine[21]. Palou et al. similarly showed excellent tolerability and little absorption with a single dose of gemcitabine immediately after TUR[22].

The chemoablative activity of intravesical gemcitabine was recently reported in a marker lesion study in patients with papillary Ta T1 grade 1-2 disease[23]. With a sample size of 39 patients, 2000 mg of gemcitabine in 50 saline given weekly for 6 weeks was able to ablate 56% of the marker lesions completely.

All reports published thus far confirm the low systemic absorption of gemcitabine, the good tolerability with minimal and systemic toxicity, and, more importantly, its efficacy as an intravesical agent, even in heavily pretreated patients. This agent warrants further investigation in a large cohort of patients especially to determine the long-term durability.

Another potential chemotherapeutic drug that is still in the intravesical formulation and testing stage is paclitaxel. Its high lipid solubility creates logistic difficulties for efficient intravesical delivery. However, use of dimethyl sulfoxide or bioadhesive polymer microspheres may circumvent this difficulty[24,25]. *In vitro* experiments have confirmed that even a brief 2-h exposure of bladder cancer cells has significant anticancer potency[26].

Intravesical docetaxel has exhibited minimal toxicity and no systemic absorption in a phase I trial that included patients with SBC resistant to standard intravesical therapy[27].

## FLUORECENCE

Fluorecence detection with 5-aminolevulinic acid (ALA) has been evaluated in a prospective randomized trial by Sachs et al.[28]; 115 patients with suspected superficial bladder cancer were randomized to undergo TUR either under standard white light or underviolet blue light after prior instillation of 50 ml of a 3% ALA solution. A second look TUR was performed at 6–8 weeks and patients were followed up for 23–61 months.

The results showed that the rate of tumor recurrence was significantly decreased in the ALA group. This advantage could be shown even after 5 years. Time to first recurrence was significantly increased and tumor progression was decreased after ALA-TUR.

## ORAL DRUGS FOR PROPHYLAXIS OF SUPERFICIAL BLADDER CANCER

A *Lactobacillus casei* preparation (BPL) is an antiflatulent that has an immunostimulant activity. The oral administration of BPL has been reported to be effective for preventing recurrences of superficial bladder cancer.

Koga et al.[29] in a randomized, controlled, phase III study, treated 102 patients with Ta-T1 TCC of the bladder with TUR followed by eight instillations of epirubicin (30 mg/80 ml) and 100 patients with the same schedule with the addition of oral administration of BPL for 1 year. Both groups received one single instillation of epirubicin within the first 6 h after TUR. The results showed that the 3-year, nonrecurrence rate in the group of patients who received oral BPL and epirubicin was 71 vs. 58.4% in the group who received epirubicin only. This difference was statistically significant. The authors concluded that intravesical epirubicin in combination with oral BPL was significantly better than epirubicin alone in reducing recurrence in Ta-T1 TCC of the bladder[29].

## NEW IMMUNOSTIMULANTS

While BCG retains its role as the most effective intravesical agent, the liability of a live organism capable of causing a life-threatening, septic infection has encouraged a search for an equally effective, but less toxic alternative. The most well-developed, new immunostimulant on the horizon is Mycobacterial Cell Wall-DNA Extract (MCC). Prepared from *Mycobacterium phlei*, the compound contains a mixture of immunostimulatory mycobacterial DNA attached to antigenic cell wall. In the recently released phase II trial results, either a 4- or 8-mg weekly dose for 6 weeks was well tolerated with a complete response rate in CIS+/-T1G3 patients of between 27–38% at 12 months[30]. Further trials are in preparation.

## THE FARTHER FRONTIER

Efforts to develop gene therapy approaches are still in the early stages, though proof-of-principle has been established in animal models and limited phase I clinical trials[31,32].

## CONCLUSIONS

There are several developments in superficial bladder cancer therapy that are likely to emerge as practical alternatives in the near future. Among the most developed are the new technology machines using microwave or electrical energy to facilitate chemotherapeutic drug transport into the bladder wall. Among the new chemotherapeutic drugs, intravesical gemcitabine is showing promising activity, while taxane formulations lag a bit behind. The prospect for combined or sequential chemo- or chemoimmunotherapy remains enticing, but underdeveloped. New immunostimulants will continue to be sought as alternatives to BCG. Further out on the horizon are more novel approaches including gene therapy, active cell-based immune therapy, and signal targeting small molecule drugs.
